# From pristine aragonite to blocky calcite: Exceptional preservation and diagenesis of cephalopod nacre in porous Cretaceous limestones

**DOI:** 10.1371/journal.pone.0208598

**Published:** 2018-12-19

**Authors:** Katarzyna Janiszewska, Maciej Mazur, Marcin Machalski, Jarosław Stolarski

**Affiliations:** 1 Institute of Paleobiology, Polish Academy of Sciences, Twarda, Warsaw, Poland; 2 University of Warsaw, Department of Chemistry, Laboratory of Electrochemistry, Pasteura, Warsaw, Poland; Universitat Bremen, GERMANY

## Abstract

Aragonite (along with calcite) is one of the most common polymorphs of the crystalline calcium carbonate that forms the skeletal structures of organisms, but it has relatively low preservation potential. Under ambient conditions and in the presence of water, aragonite transforms into calcite, the stable polymorph. Aragonite is also more soluble therefore, in water-permeable siliceous limestones (opokas) that are typical of Upper Cretaceous deposits of Poland and Ukraine, the primary aragonitic skeletons are either entirely dissolved and found as moulds and casts or transformed into secondary calcite, whereas the primary calcitic shells remain well preserved. Contrary to the common notion of the lack of aragonite in such porous carbonate deposits, we show that relics of aragonite can be preserved as a nacreous lining on cephalopod moulds or as thin, lenticular structures entrapped in neomorphic calcite. Based on the observed intermediate steps of aragonite alteration, we propose an extended model of nacre diagenesis. Among the originally aragonitic biota, only nautilids and ammonites have retained relics of pristine skeletons. Such selective preservation of only some aragonitic structures (nacre but not the prismatic aragonitic layers) points to the role of microstructural and biochemical differences between cephalopod shell layers that may set a threshold for the dissolution, dissolution/precipitation or preservation of original biomineral structures.

## Introduction

The aragonite skeletons of organisms are generally thought to have little chance for pristine preservation in the fossil record because, under the ambient conditions, aragonite is a metastable polymorph of calcium carbonate that, in the presence of water transforms into the most stable calcite. During laboratory experiments, synthetic aragonite in a solution at a temperature of 45°C converts to calcite within a few hours [1]. In nature, biogenic aragonite also undergoes polymorphic transformation relatively quickly [2,3]. The change in density between the original and secondary phases (2.94 g/cm^3^ of aragonite vs. 2.71 g/cm^3^ of calcite, respectively) and the dissolution/precipitation processes that occur along the diagenetic reaction front most commonly blur or entirely destroy the original microstructure of the skeleton. Aragonite is also more soluble in aqueous solutions than low-Mg calcite: 4.36 (±0.12) vs. 6.65 (±0.12) × 10^−7^ mol^2^ kg^−2^, respectively [4,5]. This results in preferential dissolution of the less-stable aragonite (as well as high-Mg calcite) during early diagenesis and the common preservation bias against aragonite fossils in the geological record [6–8]. Such dissolution of biogenic calcium carbonates can also be facilitated by a drop in pH in the local depositional environment as the animal body decays (both the soft tissue inside the shell and the interskeletal organic matrix) due to acidic byproducts, such as sulfuric acid (H_2_SO_4_), CO_2_(aq) and volatile fatty acids, produced from microbial metabolism and autolysis [9]. The calcifying microbial mats provide a broad spectrum of CaCO_3_ dissolution/precipitation processes that result from, e.g., changes in the intensity of microbial metabolism (shifts between dissolution and precipitation), CaCO_3_ dissolution induced by aerobic respiration, sulfide oxidation and fermentation [10]. The microbial activity also influences the chemical composition of the sediment during early diagenesis [11]. The dissolution of skeletal aragonite in the shallow subsurface sediments can by influenced by microbial degradation of sedimentary organic matter (aerobic oxidation of organic matter, oxidation of reduced byproducts and H_2_S, sulfate reduction or anaerobic methane oxidation) [12,13]. In the subsequent stages of burial history that usually include meteoric diagenesis, the aragonite fossils are again exposed to dissolution or replacement by calcite. Consequently, aragonite skeletons are frequently either removed from the fossil record or their original mineralogy, microstructure, and geochemical signatures are altered.

Although aragonitic remains seem to have low preservation potential, original biogenic aragonite has been described from as early as Paleozoic strata (ca. 450 Mya [14]). The aragonite nanocrystals associated with the organic globules in stromatolites date back even earlier, ca. 2.7 Gya (see [15]). Kennedy and Hall [16] suggested that these are hydrophobic protective layers derived from the breakdown of skeletal organic matrices that slow the process of CaCO_3_ phase transition in biogenic crystals. The importance of organic matter for the preservation of skeletons in the fossil record is evident when comparing biogenic vs. geological and synthetic aragonites. Experiments have demonstrated that, synthetic aragonite rapidly transforms into calcite in the presence of water, whereas biogenic aragonite can survive much longer [16].

**Fig 1:**
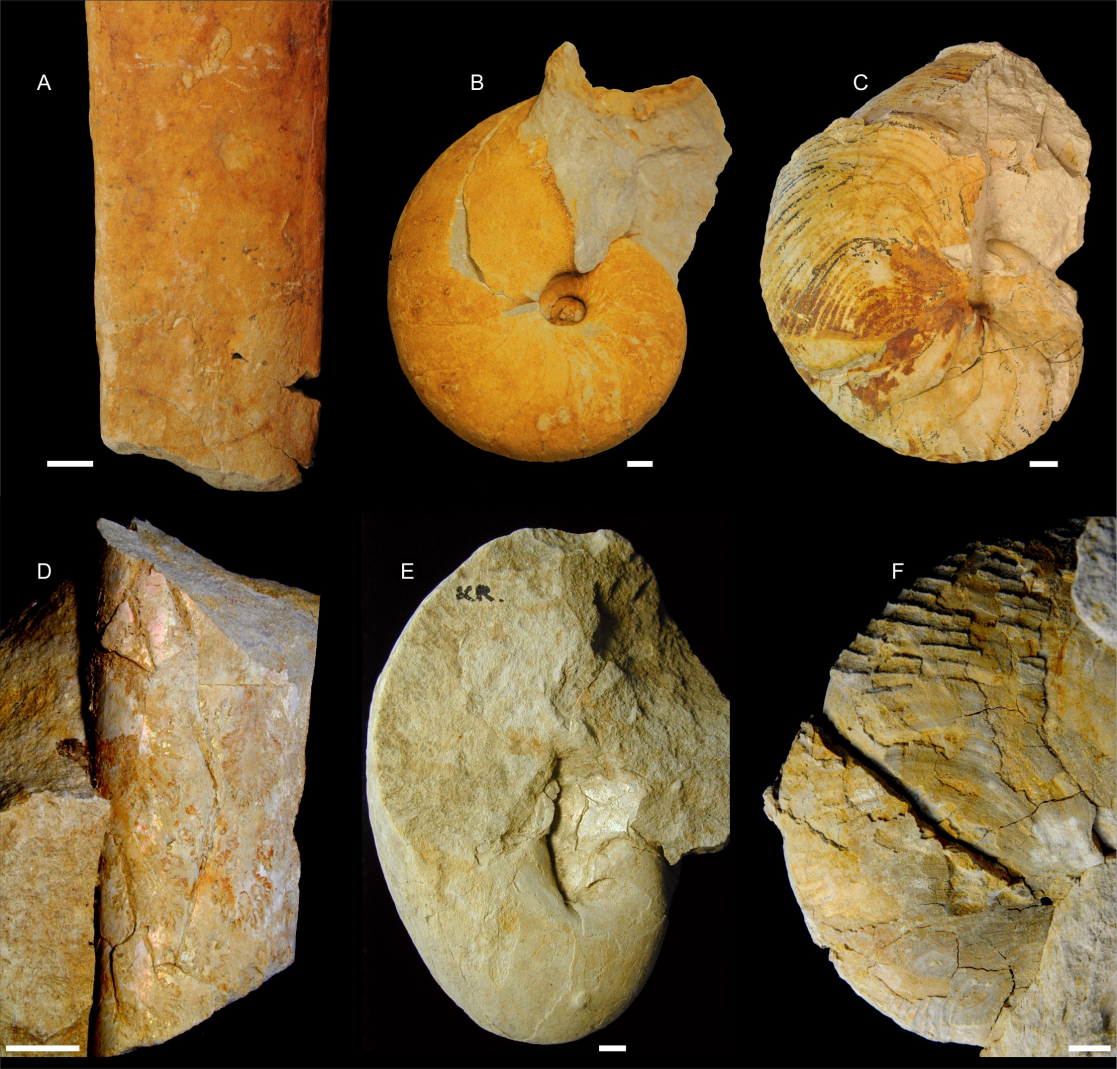
Two modes of cephalopod preservation in the upper Cretaceous siliceous limestones of eastern Poland. The majority of originally aragonitic cephalopod shells are typically dissolved and preserved as moulds covered with orange-brown iron deposits, sometimes with well-discerned ornamentation (upper row: A-C). In contrast, the specimens studied herein retain parts of originally aragonitic shells, preserving lustrous surfaces formed by nacre (lower row: D, E). (D) Fragments of ammonite nacreous layer show typical iridescence; note void between the specimen and the host rock, indicating that the outer part of the shell (formerly thicker) was dissolved after lithification of the sediment. (E) Shiny fragment of nacreous layer preserved on the umbilical part of nautilid internal mould. (F) Nautilid mould still covered with large fragments of original ribbed shell wall (compare with C–internal mould of the same species) that retain remnants of aragonite nacre layers (see Fig 4). (A) *Baculites anceps*, Nasiłów (ZPAL Am. 12/360). (B) *Cymatoceras intrasiphonatum*, Nasiłów (ZPAL N.III/125). (C) *Cymatoceras*? *patens*, Piotrawin (ZPAL N.III/159). (D) *Baculites* sp., Krasnobród (ZPAL Am. 12/1375). (E) *Eutrephoceras vastum*, Krasnobród (ZPAL N.III/219). (F) *Cymatoceras*? *patens*, Krasnobród (ZPAL N.III/224). The age of the specimens from Nasiłów is late Maastrichtian, from Piotrawin late Campanian, and from Krasnobród either latest Campanian or earliest Maastrichtian. Scale bar = 10 mm.

The preservation of aragonite in the paleontological record is also favored by (relatively) low temperatures and isolation from water, e.g., by bitumen impregnation [17] or rapid burial in impermeable sediments [18]. Although the occurrence of aragonite fossils is quite common in Cenozoic deposits, the preservation potential of aragonite decreases with geological age (see [18]). The aragonite fossils of Mesozoic and (only exceptionally) Paleozoic age are only found when enclosed by argillaceous rocks or other impermeable deposits, so the highly porous siliceous limestones (opokas) typical of Upper Cretaceous deposits of central Europe (see [19,20]) are not considered a suitable environment for the preservation of pristine aragonite. To date, no fossils with preserved original aragonitic shells have been reported from these deposits; the formerly aragonitic shells typically occur as moulds (Fig 1A–1C; see also [21–23]) or entirely transformed (calcitized) specimens. This is in stark contrast to primarily calcitic skeletons or their parts (e.g., belemnite rostra), which are typically morphologically well preserved (e.g., [24]). Unexpectedly, in some Upper Cretaceous siliceous limestone sections in Poland and Ukraine (S1 Fig), we found nautilid and ammonite moulds with iridescence on their surfaces (Fig 1D and 1E) characteristic of original lustrous, aragonitic layers of mother-of pearl [25,26]. In this study we have confirmed aragonite preservation, and based on a larger collection of fossils from the studied sections, we have illustrated a broader spectrum of preservation of originally aragonitic structures from almost intact layers of nacre, relics of aragonite in neomorphic calcite, to skeletons entirely transformed into blocky calcite. These different preservation states are documented herein by various analytical techniques that allowed us to complement the model of cephalopod nacre diagenesis proposed in the 1980s (e.g., [27,28]).

## Materials and methods

### Materials

The fossils of cephalopods (*Eutrephoceras* and *Cymatoceras*? nautilids and *Baculites* sp. heteromorph ammonites) with aragonite remnants were collected from two Upper Cretaceous (Campanian/Maastrichtian) sections exposed at Krasnobród in eastern Poland and at Potelych in western Ukraine. Comparative materials (cephalopods, bivalves, gastropods and other calcium carbonate skeletons) came from several Upper Cretaceous localities in Poland and Ukraine (S1 Fig). The detailed list of specimens treated and illustrated in the present study is provided in the Supporting Information (S1 Tab). The specimens studied in this paper were subject to destructive analyses, and the resulting thin sections and skeletal fragments attached to microscope stubs are housed at the Institute of Paleobiology, Polish Academy of Sciences, Warsaw (ZPAL).

The aragonite-bearing sections are located in the Roztocze area, which is a range of hills extending from the area of the town of Kraśnik (southeast Poland) to the town of Lviv (western Ukraine) with the Campanian and Maastrichtian deposits outcropping in several places (see [24] for the stratigraphy of key sections in Poland and [29] for the same for the Ukrainian part of Roztocze). No specific permissions were required to access these locations, which are not part of a protected area.

Locality Krasnobród (50°32’58”N, 23°12’14”E) is a large abandoned quarry situated in the town of Krasnobród, ca. 500 meters north-east of the town center ([30]: fig. 1c). The quarry section exposes approximately 15 meters of siliceous limestones that alternate with thin marly intercalations; the section yielded abundant marine macrofossils including ammonites, nautilids (aragonite relics were only found in fossils of these two groups), belemnites and bivalves as well as well-preserved remains of leaf flora transported from the nearby land area [31]. Stratigraphically, the Krasnobród section spans the Campanian/Maastrichtian boundary, representing the uppermost Campanian *Belemnella inflata* Zone in its lower part and the lowermost Maastrichtian *Belemnella vistulensis* Zone [24] in the upper one. In terms of the inoceramid zonation of Walaszczyk et al. [32,33], the lowermost portion of the quarry section belongs to the *“Inoceramus” costaecus* Zone, its major component representing the “*Inoceramus*” *redbirdensis* Zone, the upper part of which comprises the Campanian/Maastrichtian boundary [24,32,33]. No data on the precise location of our specimens in the section are available, so they may be of latest Campanian or earliest Maastrichtian in age.

Locality Potelych (Potylicz in the old Polish literature) (53°13’N, 23°33’E) is a large, abandoned quarry situated ca. 500 meters north-west of the village Potelych, near the town of Rava Russkaja, not far from the Polish-Ukrainian border. The section at Potelych exposes ca. 8 meters of siliceous limestones with relatively abundant remains of ammonites, nautilids, bivalves, gastropods, echinoids and land flora [31]. The aragonite relicts were only found within cephalopod fossils. Inoceramids allow the Potelych succession to be assigned to the “*Inoceramus*” *costaecus* Zone [34], which represents the upper Campanian in the scheme of Walaszczyk et al. [32,33].

### Methods

#### Transmitted light microscopy (TLM) and scanning electron microscopy (SEM)

Images were obtained from transverse and longitudinal thin sections of specimens or partly broken specimens observed under a conventional transmitted light microscope (TLM) and scanning electron microscope (SEM), respectively. Thin (ca. 30-μm thick) sections of specimens were observed in transmitted and polarized light and photographed with a Nikon Eclipse 80i transmitted light microscope fitted with a DS-5Mc cooled camera head. For SEM, skeletal fragments were placed on stubs with double-sided electrically conductive tape and sputter-coated with conductive platinum or carbon film. The selected fragments of thin sections and fractured specimens were slightly etched with formic acid (1%, 15 s) before observation with SEM. Analyses were performed using a Phillips XL20 scanning electron microscope at the Institute of Paleobiology, Polish Academy of Sciences and a Zeiss Merlin field emission scanning electron microscope at the Faculty of Chemistry, University of Warsaw, Poland.

#### Raman microscopy

The mineralogical composition of thin-sectioned specimens was analyzed by Raman confocal microscopy. Raman maps of the samples were collected at the Department of Chemistry, University of Warsaw with a LabRAM 800 HR Raman spectrometer (Horiba Jobin Yvon) coupled to an upright optical microscope (Olympus BX41). The spectra were excited with a diode-pumped Nd:YAG laser (Excelsior-532-100, Spectra-Physics) operating at 532.3 nm (ca. 2 mW of power on the sample). The spectrometer was equipped with an LPF Iridia edge filter, a 600-groove mm^-1^ holographic grating and a 1024 x 256-pixel Peltier-cooled Synapse CCD detector. The maps were collected with a MPLN 100x objective at a 1-s integration time and a pixel size of 1 μm x 1 μm.

Calcium carbonate polymorphs reveal bands assigned to internal mode vibrations of CO_3_^2-^ ions (1085 cm^-1^) and rotational and translational lattice modes. Bands characteristic of calcite are found at 281 cm^-1^ and 154 cm^-1^, whereas they are located at 205 cm^-1^ and 153 cm^-1^ for aragonite. The analysis of the maps and modeling of the distribution of CaCO_3_ polymorphs was done with Labspec 5.45 software (Horiba Jobin Yvon). The modeling algorithm was based on correlation fitting of known reference spectra using the direct classical least squares method.

#### X-ray diffraction

X-ray diffraction was used to determine the mineralogy (the presence of aragonite in the iridescent coating) of the two specimens. Analyses were performed at the Institute of Geological Sciences (Warsaw) with a CGR-INEL diffractometer equipped with a cobalt cathode X-ray lamp (Siemens) and focusing goniometer with transmissions optics for Debay-Scherrer powder preparations.

#### Cathodoluminescence microscopy (CL)

Cathodoluminescence microscopy is one of the techniques used to evaluate the preservation state of originally aragonitic fossils. As shown in studies of recent specimens, the intact shells of nautilids exhibit two types of weak luminescence, yellow-greenish or blue [35]. The orange luminescence within originally aragonitic fossil shells usually correlates with calcitized regions of a skeleton that were diagenetically enriched in manganese [35]. The bright orange luminescence can also be excited by Mn incorporated in the calcite crystals *in vivo*, e.g., in the growth layers of originally calcitic, non-altered skeletons [36], or it may represent places of shell repair [37]. However, based on the known primary aragonitic mineralogy of the shells of Cretaceous nautiloids and ammonites and the lack of correlation between the spatial delineation of luminescence and growth banding, we interpreted orange and red regions on CL images as affected by diagenesis (calcitized). In some fossil skeletons (see, e.g., [38]), the less-altered areas (containing remnants of aragonite) might be nonluminescent but easily distinguishable from the bright orange background formed of secondary calcite. The hot cathode microscope HC1-LM at the Institute of Paleobiology, Polish Academy of Sciences was used to trace possible diagenetic alteration of the studied skeletons. Thin sections were sputter coated with carbon prior to examination by CL. An electron energy of 14keV and a beam current density of 0.1 μA/mm^2^ were used for CL microscopy visualization.

## Results

The originally aragonitic parts of the cephalopod shells studied herein were preserved as follows: (i) an iridescent lining composed of unaltered or only slightly changed nacreous layer, (ii) fragments of calcitized skeletons that preserved relicts of aragonite nacre, or (iii) completely recrystallized shells composed of blocky calcite. Below, we describe examples of each type of preservation.

### Nacreous layer with (almost) intact structure [i]

**Fig 2:**
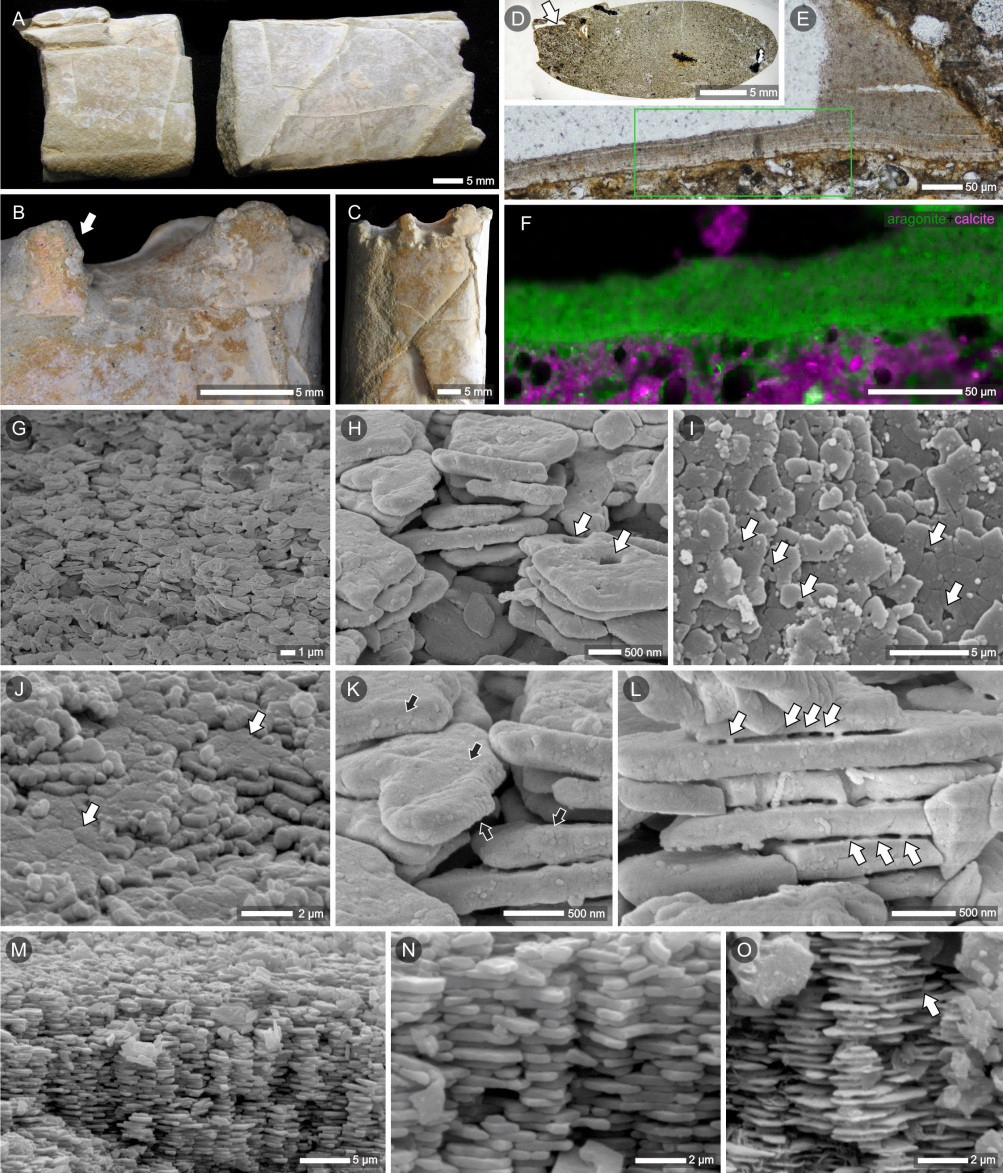
Nacre preservation in the late campanian ammonite *Baculites* sp. from Potelych (Potylicz), western Ukraine. (A) Internal mould covered with a white coating and (B, C) close-ups of the suture pattern and fragment of iridescent nacre (B, arrow). (D, E (enlarged)) Thin section of the specimen in transmitted light; note the layered structure of the shell (the green frame outlines the region shown in F). (F) Aragonite (green) mineralogy of remnants of the nacre layer and calcite of internal mould deposit (magenta) visualized in a micro-Raman map. (G, H (enlarged)) FESEM micrographs of the white coating of the mould formed of small (ca. 2–3 μm in diameter, ca. 300 nm thick) polygonal tablets. The rounded cavities (arrows in H, I) in the tablets are traces of central organic accumulations. These holes also penetrate underlying tablets of nacre, as it is particularly well seen in the top view (I, arrows). (I) SEM micrograph showing parallel layers of thin polygonal tablets that form the iridescent surface of the shell. (J) Oblique view of shell surface showing that some of the tablets have preserved their division into sectors (SEM image). (K) FESEM close-up of (H) showing granular texture of aragonite tablets (black arrows, see also J and L). The rounded granules (ca. 60–90 nm in diameter) visible on the surface of tablets are remnants of the primary nanostructure of aragonite tablets. (L) FESEM close-up revealing the presence of small vertical structures (ca. 70 nm in diameter) that connect superposed tablets of nacre and resemble mineral bridges observed in the nacre of modern mollusks; see [43]). (M-O) SEM micrographs of fractured fragments of nacreous layer formed of vertical stacks of aragonite tablets. Occasionally, within the same specimen, the original nacre tablets can be slightly dissolved (reduced tablet thickness), which emphasizes the columnar microstructural pattern (O) and vertical connections (bridges) between tablets (O, arrow; compare with L). (A-O) Specimen ZPAL Am. 12/1374.

*Baculites* sp. from Potelych (ZPAL Am. 12/1374)—a thin white coating covers the entire surface of the mould (Fig 2A–2C). The thickness of this layer is ca. 40 μm in thin section (Fig 2E). SEM micrographs of a smooth iridescent fragment of a surface show small polygonal tablets closely adjacent to each other (Fig 2I and 2J) and horizontally arranged in layers. Tablets are ca. 3 μm in diameter and ca. 0.3–0.4 μm in thickness. SEM close-up (Fig 2J) shows that the tablets are composed of sectors as previously observed in recent and well-preserved Callovian shells (see [39] fig. 6a-c; [40] fig. 8.7a). Some of the plates have small rounded cavities (arrows in Fig 1F and 1H) resembling traces of central organic accumulation observed in nacre tablets of nautilids and ammonites ([39] fig. 5c; [40] fig. 8.7a; [41]; [42] fig. 2). FESEM micrographs revealed that the aragonite tablets have a granular texture (Fig 2K); rounded granules are ca. 60–90 nm in diameter and most likely are remnants of the original nanostructure of nacre tablets (see [39]). Layers of nacre run parallel to the surface. The cross-sections of the shell wall show columnar arrangement of tablets (Fig 2M and 2N) typical of cephalopod and gastropod nacre,and dissolution emphasized this pattern in more altered fragments (tablets are thinner and smaller in diameter; Fig 2O). The small (ca. 70–100 nm in diameter) vertical structures connect superposed tablets of aragonite (Fig 2L and 2O) resembling structures described in modern mollusks as mineral bridges [43]. The Raman microscopy analysis confirmed the presence of aragonite in an iridescent layer (Fig 2F); this is the best-preserved specimen with the largest non-altered surfaces of nacreous layer.

**Fig 3:**
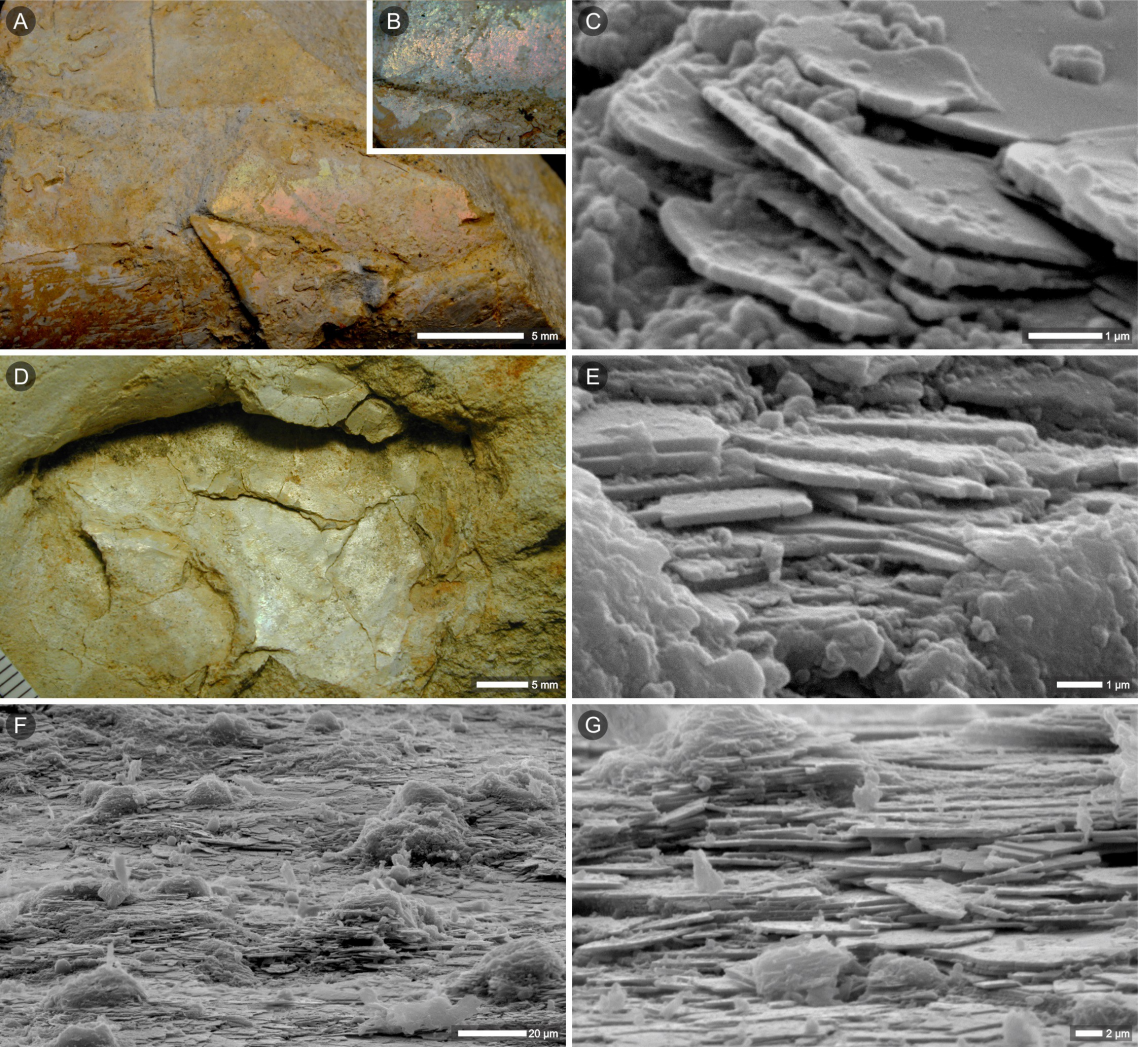
Nacre preservation in the latest campanian or earliest Maastrichtian cephalopods from Krasnobród (Poland). (A-C) Internal mould of ammonite *Baculites* sp. covered with fragments of the original mother of pearl: macrophotograph (A) and close-up (B) of iridescent coating; (C) SEM micrograph (C) of the mould surface with remnants of the nacreous layer. **(**D-G) Internal mould of nautilid *Eutrephoceras vastum* (D) close-up showing lustrous fragment of the shell preserved in the central part of the specimen; (E-G) SEM micrographs of the nonetched part of the specimen (D) showing superposed layers of nacreous tablets. (A-C) Specimen ZPAL Am. 12/1375, (D-G) specimen ZPAL N.III/219.

*Baculites* sp. from Krasnobród (ZPAL Am. 12/1375)—mould covered with a thin (ca. 10–15 μm) iridescent layer (Figs 1D, 3A and 3B). The thickness of the primary shell (prior to dissolution) is estimated at ca. 600 μm based on the void space between the mould and host rock. When observed in SEM, iridescent surface of *Baculites*, appeared to be formed of superimposed rounded tablets ca. 0.25-μm thick (Fig 3C). The lining was too thin to perform thin sections and subsequent micro-Raman mapping of the iridescent layer. Instead, the occurrence of aragonite in this specimen was revealed by XRD analysis performed on a scratched sample of glossy nacre (S2A Fig).

Nautilid *Eutrephoceras vastum* (Kner, 1848)[44] from Krasnobród (ZPAL N. III/219)—fragmentarily preserved glossy surface covers umbilical portion of the mould (Figs 1E and 3D) but is also present on the outer whorls. SEM micrographs of nonetched samples of this coating show superimposed polygonal tablets (10–15 μm in diameter) that form layers parallel to the shell surface (Fig 3F and 3G). In cross-section, rounded tablets are 0.25–0.3-μm thick (Fig 3E) and composed of aragonite (XRD analysis of scratched lustrous shell fragments of this specimen; S2B Fig).

### Nacre relicts [ii] in neomorphic calcite [iii]

**Fig 4:**
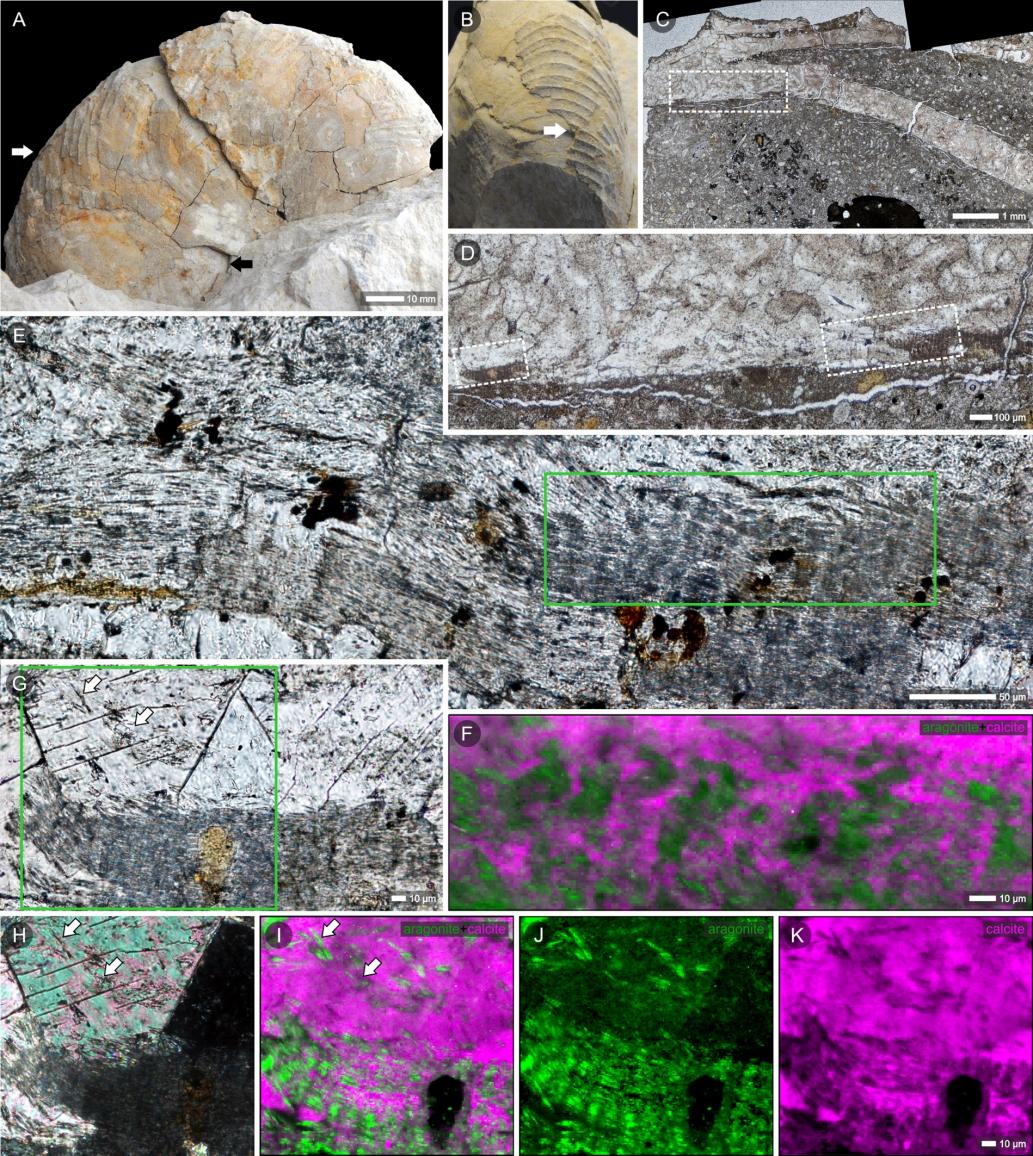
Relics of nacre in diagenetically altered shell of the latest campanian or earliest Maastrichtian nautilid *Cymatoceras*? *patens*, Krasnobród (Poland). Macrophotographs of specimen in lateral (A) and ventral (B) views; white arrows show sectioned fragment (C-K), black arrow (in A) show sampled region illustrated in Figs 5A, 5B and S3. (C) Thin section of the shell wall and septum (plane perpendicular to shell ribs) in transmitted light (dashed frame in C indicate region enlarged in D); (D-E, G) close-ups of a lower (inner) part of the septum with layer composed of thin lamellae arranged parallel to the septal plane (dashed frames in D indicate E and G enlargements); the green frames in E and G indicate regions analysed with micro-Raman (F and I-K, respectively). (F) Raman microscopy mapping of CaCO_3_ polymorph distribution; dark banding visible in transmitted light (E) corresponds to aragonite (green) areas in Raman map. Magenta represents regions enriched in calcite. (G-K) Thin-sectioned fragment of the septum with darker, laminated zone (remnants of nacreous layer) that interfinger with strongly altered region composed of blocky calcite (G, transmitted light; H, fragment of G in polarized light); (I-K) Micro-Raman images showing that small enclaves of aragonite (green) are entrapped in calcite crystals (magenta), (arrows in G-I); the linear arrangement of aragonite tablets is still discerned, remnants of nacreous layers cross crystal boundaries and calcite cleavage lines (G-I, arrows). (A-K) Specimen ZPAL N.III/224.

Nautilid *Cymatoceras*? *patens* (Kner, 1848)[44] from Krasnobród (ZPAL N. III/224)—fragmentarily preserved, but the thickest of the studied shells; ornamentation still present; iridescence not observed (Fig 4A and 4B). The thickness of the shell (measured from fragment embedded in the host rock) is ca. 1.8 mm. The thin section perpendicular to the ribs revealed the fragment of nautilid septum (Fig 4C). The shell wall is now formed of coarse crystals of calcite (calcite cleavage lines are seen in optical microscopy; Fig 4C) that do not contain any hint of which layers of primary shell are preserved, i.e., diagenetically altered prismatic and nacreous layers or only nacreous.

**Fig 5:**
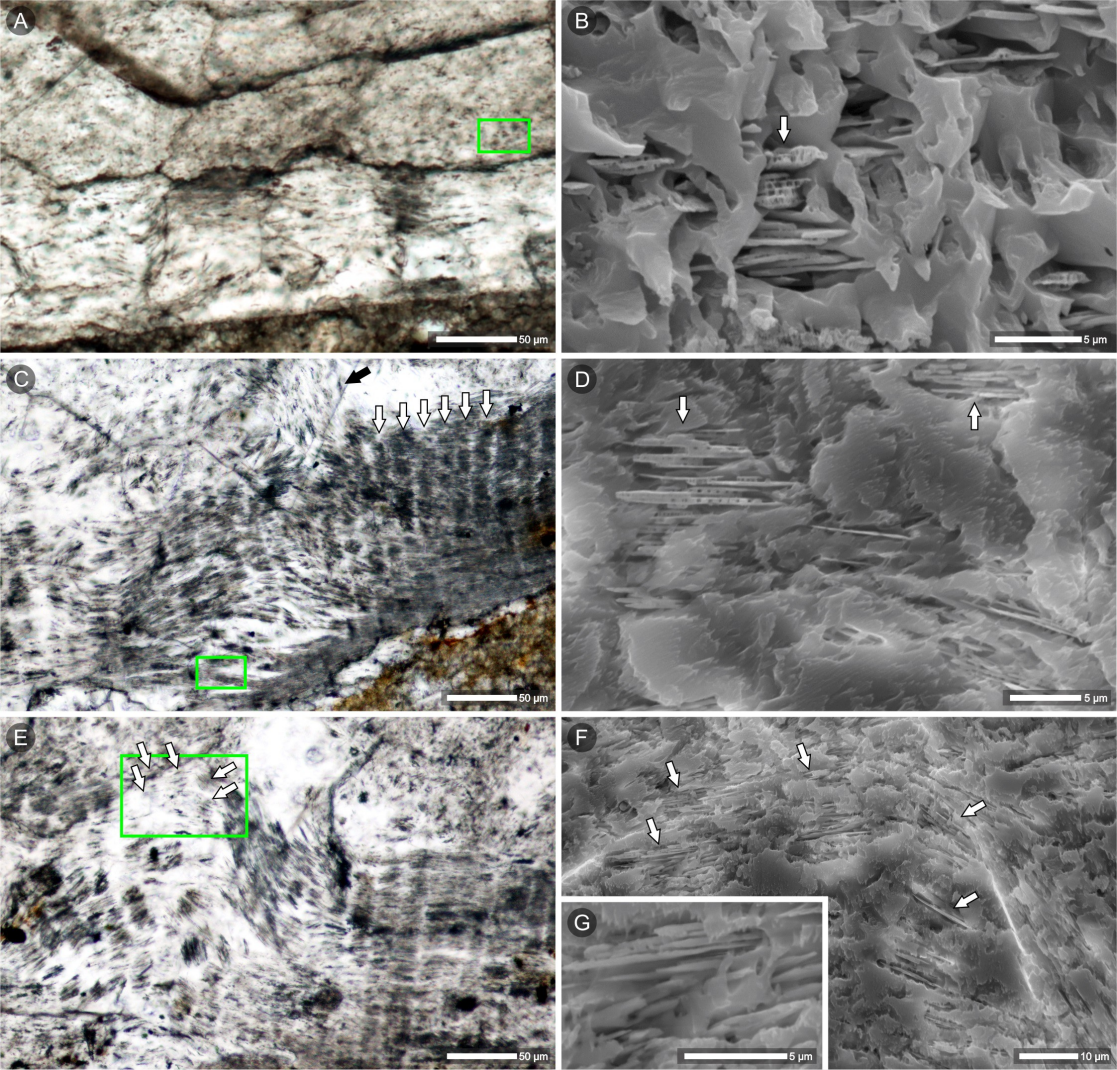
Relics of nacre in diagenetically altered shell of the latest campanian or earliest Maastrichtian nautilid *Cymatoceras*? *patens*, Krasnobród (Poland). Thin sections of the shell wall (A-B) and septum (C-G) in transmitted light (left column) and slightly etched in SEM (right column); the green outlines in transmitted-light images indicate regions enlarged in SEM images. Darker regions (transmitted light, arrows) correspond to zones containing aragonite tablets stacked between crystals of neomorphic calcite (SEM images). Remnants of nacre tablets show the original columnar arrangement (B, C, white arrows) but thin, lenticular shapes (B, D, G). The regularly arranged pores visible within those stacks (B, D arrows) most likely result from selective etching of tablets and reflect heterogenous structural and biogeochemical composition of nacre tablets (see [45,46]). Thin aragonite tablets, when observed in thin section, are visible as linear impurities and cross crystal boundaries of secondary calcite (C, black arrow). Even in strongly altered fragments of the shell, parallel layers of nacre tablets are still discernible (D, F); E—development of sparitic crystals results in undulations of originally flat nacre layers. (A-G) Specimen ZPAL N.III/224.

The lower part of the preserved septum is formed of thin lamellae (each ca. 0.5-μm thick; Fig 4D, 4E and 4G). Superposed lamellae are parallel to the septal surface and form a brownish layer that is well distinguished in transmitted light. Further from the septal edge, this laminated layer gradually passes into coarse crystals of calcite (Fig 4D and 4E). In some regions, the laminated layer interfingers with coarse crystals of calcite with distinct cleavage lines (Fig 4G and 4H). Smaller fragments of disintegrated lamellae are visible as inclusions within larger crystals of calcite (Fig 4G and 4H). In thin sections, the layer shows a pattern of alternating dark and light bands arranged in the direction normal to the septal plane (Figs 4E, 5C, 5E and S4C and S4E). Raman microscopy maps (Fig 4F) show that this part of the septum has mixed calcitic/aragonitic mineralogy and areas darker in transmitted light images correspond to regions enriched in aragonite (green in the Raman-based images, Fig 4E and 4F). The micro-Raman mapping also confirmed the presence of aragonite inclusions enclosed in calcite crystals (green islands within the magenta areas of calcite; Fig 4I and 4J). In addition to the laminated regions described above, most of the septum is composed of calcite crystals, some of which show dark banding (linearly arranged brown impurities) that cross the crystal boundaries (Figs 4G and 5C, black arrow).

More details about the relationships between the primary (nacre) and secondary (calcite) components of the shell can be seen from SEM of slightly etched thin sections (Fig 5D and 5F). Thin aragonite laths, which are the remnants of nacre tablets, are preserved in the lowest (innermost) part of the septum (similar “lath-shaped inclusions” of aragonite in neomorphosed shells of Jurassic bivalves were described by Hendry et al. [47]). Larger fragments of columnar stacks of tablets may occasionally occur as enclaves surrounded by calcite (Fig 5D). In a more altered region of the shell, single laths of aragonite can be observed; entrapped within neomorphic calcite, they still show traces of their original, parallel arrangement and periodic spacing between the layers of nacre (Fig 5F). The geochemical (EDS) analysis of the ZPAL N. III/224 section, where aragonite laths were identified (S5 Fig 1–5), showed a slightly higher concentration of magnesium in the aragonite compared to the secondary calcite (Mg/Ca molar ratios: 11.9–12.9 mmol/mol vs. 6.1–10.9 mmol/mol, respectively). The strontium (which is typically enriched in aragonite) was actually only detected in the aragonite in the better-preserved part of the septum (Sr/Ca = 22.8 mmol/mol).

The thin section of the umbilical part of the specimen (black arrow in Fig 4A) shows dark banding parallel to the shell surface that is most noticeable in the inner part of the shell (lower part of the section; Figs 5A and S3A, S3C and S3G). Regions darker in transmitted light correspond to cross-sections of aragonite tablets, as shown in SEM images of the same, slightly etched section (Fig 5B). Aragonite tablets are very thin (200-300-nm thick) and lie parallel to the shell surface. In other parts of this section, the shell is entirely diagenetically altered with a typical network of crystal faces and cleavage lines of newly formed calcite (S3H Fig). Furthermore, most of the surface of the same section (black arrow in Fig 4A) exhibits bright luminescence in CL (S3B Fig). The lack of luminescence of the innermost part of the shell (white arrow in S3B Fig) could suggest that this region is less altered than the middle, bright luminescent part. However, regions of different luminescence have similar distributions of aragonite relics. SEM and transmitted light images exhibit thinned tablets of nacre enclosed in neomorphic calcite (S3E–S3G Fig), which is confirmed by Raman microscopy maps showing that thin aragonite inclusions are embedded in a calcite matrix in both regions (S3D Fig). The lack of clearly different aragonite signals in the bright luminescent part of calcitized shell most likely results from suppression of the aragonite luminescence by the bright luminescence of adjacent diagenetically altered regions.

Higher magnifications of the inner part of the septum (arrows in S4 Fig) show delicate banding arranged perpendicular to the septal surface (S4F Fig). Alternating dark and bright bands in CL images correspond to those observed in transmitted light (S4E Fig). This periodic banding pattern is well discerned in Raman microscopy maps of the same area (aragonite-enriched areas separated by calcite, Fig 4E and 4F) and resembles the columnar arrangement of tablets in the nacreous layer observed with SEM (e.g., Figs 2M–2O and 5D). Consequently, regular alteration between luminescent and nonluminescent regions in CL images most likely reflects the alternating relicts of the original microstructure and neomorphic calcite.

## Discussion

### Preservation of fossil cephalopod shells

We will further discuss the diagenetic transformations of Cretaceous specimens, but we first provide a brief overview of the major structural components of the intact ectocochleate cephalopod shell based on modern *Nautilus* and the best-preserved fossil specimens. The shell consists of four main structural components: organic periostracum, outer prismatic layer, nacreous layer, and inner prismatic layer [40]. It was once generally accepted that all three mineralized layers of cephalopod shells are aragonitic, but De Beats and Munnecke [48] provided microstructural evidence that some Siluro-Devonian orthoconic nautiloids could have outer calcitic and inner aragonitic shell layers. If the primary origin of such a calcitic shell layer is confirmed by more in-depth diagenetic studies, this finding may have major implications for cephalopod shell evolution, particularly in the context of the influence of the low Mg/Ca geochemistry of Paleozoic "calcitic seas" on shell mineralogy. Nonetheless, there is no evidence of the presence of a calcitic layer in the cephalopod shells studied here, which were formed in the Cretaceous when the ocean Mg/Ca ratio was near the lowest in the Phanerozoic [49].

The shell organization parameters may vary, e.g., the thickness of individual layers may vary in a single shell, spatial relationships may change in the ontogeny, not all layers may be present in the entire shell and the thickness of the layers may differ between the taxa [50]. With the growing size of the shell, the thickness of the nacreous layer increases, and at the end of the shell ontogeny, the outer prismatic layer constitutes a very small proportion of the shell thickness [40]. In well-preserved ammonites, the nacre comprises ~99‰ of the total shell thickness and is sandwiched between very thin prismatic layers [51]. A similar disproportion between the development of nacreous and prismatic layers occurs in modern *Nautilus*, e.g., in a specimen examined by Petrochenkov et al. [52], in which the outer prismatic layer and nacreous layer exhibited a thickness of ca. 0.02 mm and ca. 0.7 mm, respectively.

**Fig 6:**
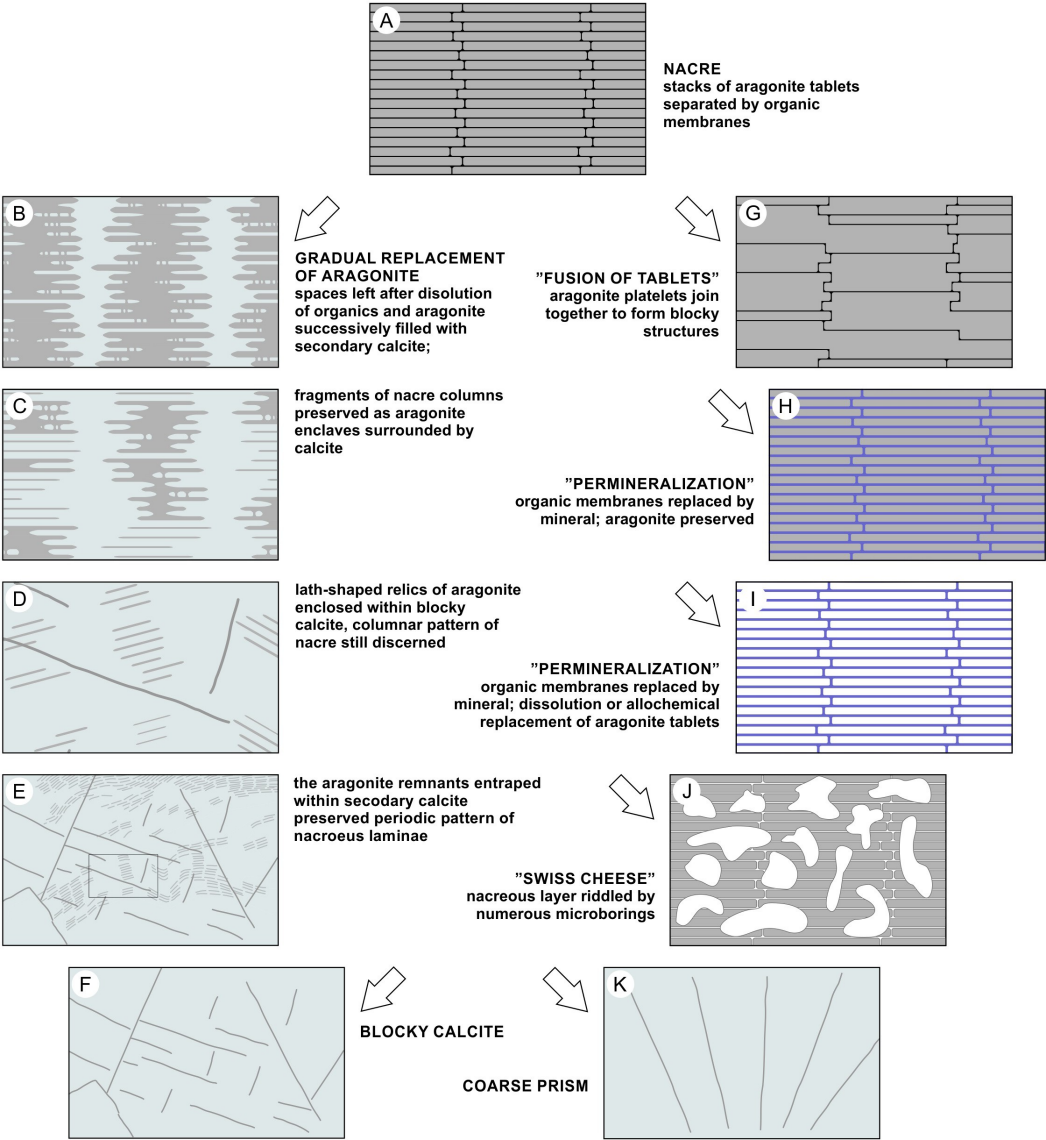
Models of structural alterations of cephalopod nacreous layer based on SEM and thin sections observations described in this paper (left column) and previous studies [28,58,59]. Arrows indicate various diagenetic pathways of aragonite. (A) Originally preserved nacre: superimposed, flat aragonite tablets form columnar units that locally may overlap. Thin organic layers separate individual tablets of nacre. (B-E) Different stages of aragonite inversion to calcite. (B) Tablets slightly lenticular in shape: degradation of organics, dissolution of aragonite, and crystallization of secondary calcite occur mainly along the boundaries between nacre columns; minor alterations occur on the lower and upper faces of tablets; dissolution exposes vertical connections between superposed tablets–mineral bridges, originally surrounded by interlamellar organics (see Fig 2L and 2O). (C) Fragments of columnar stacks of nacre tablets separated by secondary calcite. (D) Parallel lath-shaped inclusions of aragonite enclosed by calcite; the outlines of original stacks of tablets are still recognizable (enlargement of E). (E) Linearly arranged impurities (thinned tablets of aragonite) cross crystal boundaries of secondary, blocky calcite (similar impurities were previously observed by Dullo and Bandel [28] Text Fig. 14 but interpreted as relics of growth lines, not nacre tablets). (F) Blocky calcite with no traces of aragonite: primary aragonite could be neomorphically transformed to calcite or completely dissolved with resulting space filled with calcite spar. (G) Fusion of adjacent aragonite tablets that occurs after dissolution of organic boundaries between tablets (described from Plio-Pleistocene aragonite mollusks; [58]; see also [60]). (H, I) “Permineralization”–secondary minerals filled spaces between tablets of nacre (previously occupied by organic matter); (H) aragonite nacre tablets preserved [58]; (I) aragonite tablets dissolved or replaced by other mineral (see [59]). (J) Porous “Swiss cheese” structure–the nacreous layer severely damaged by bioerosion [28]. (K) Coarse prism of calcite [28].

The *periostracum* is a thin (a few μm) proteinaceous layer that covers the outer surface of the shell wall [23,40,53]. The *outer* and *inner prismatic layers* are composed of aragonite crystals arranged perpendicular to the shell surface. The prisms range from 0.2 to 0.5 μm in diameter [40], and each is outlined by organic envelopes [54]. The *nacreous layer* is formed by polygonal aragonite tablets arranged horizontally in superimposed lamellae separated by protein-rich interlamellar membranes [40]. Adjacent aragonite tablets in a lamella are separated by intertabular organic matrix [55]. Cephalopods (also gastropods, monoplacophorans and some of the primitive bivalves) have nacre composed of tablets lying one over another, forming vertical columns “with some overlap between them” (Fig 6A) [40,46,56,57] in contrast to the brick wall arrangement of nacre tablets in bivalves [46]. Superposed tablets of aragonite are connected via mineral bridges, which are vertical structures that are subcircular in cross section and 100–200 nm in diameter [43]. The aragonite tablets in mollusk nacre are 3–15 μm in diameter and approximately 0.2–0.5 μm in thickness [55,57]. The tablets in nacreous layers in the septa sometimes have diameters two-three times larger than those forming shell wall [42].

The four layers that originally formed cephalopod shells appear to have significantly different preservation potential in the fossil record. Clearly, some traces of the original outermost organic layer of shell (periostracum) only exceptionally occur in some fossil nautilids [61]; no remnants of this layer were detected in the studied specimens.

The aragonitic prismatic layer is also only rarely preserved in the paleontological record. Even in the localities considered aragonite lagerstätte, where fossils have been described as exceptionally preserved, e.g., Carboniferous Buckhorn Asphalt [62], Jurassic Łuków clays [39] or Cretaceous deposits of the Nugssuaq Peninsula in West Greenland [27] cephalopods are found without the prismatic layer. Extremely rare fossil shells with all three mineral layers preserved [63,64] suggest that the outer prismatic layer was much thinner than the underlying nacre. For example, the outer prismatic layer in Cretaceous heteromorph *Ptychoceras* was only ca. 1-μm thick (thickness of the shell ca. 50 μm; [40]) and ca. 2 μm in *Scaphites* (the entire shell wall was ca. 200-μm thick; [65]). The lack of such a thin, prismatic layer is sometimes explained as a result of “exfoliation” during excavation, or the removal of this layer could have occurred before burial, such as during postmortem transport [62]. The rapid disintegration of this layer could also be facilitated by its prismatic microstructure (for further detail, see below: “Shell microstructure and organic matrix content”). Similarly, no aragonite remnants of the outer prismatic layer were found in the cephalopods studied herein. We neither observed a primary preserved inner prismatic layer. The direct contact between the nacreous lining and the mould shows the absence of this layer in sectioned regions of both *Baculites* shells.

Only one of the examined specimens, *Cymatoceras*? *patens* from Krasnobród (ZPAL N.III/224) preserved its thick ornamented shell wall (not only the lining) that could potentially contain traces of all three mineral layers of the skeleton. However, the outer part of preserved shell is now formed of coarse calcite crystals, and there is no evidence of whether those fragments are diagenetically altered prismatic or nacreous layers.

Out of the three original mineralized layers present in modern cephalopod shells, remnants of only the nacreous layer were found in the nautilid and ammonite specimens examined herein. We identified aragonite tablets in iridescent coatings of moulds and within the septum and inner part of thick ribbed shell wall. The studied specimens illustrate a broad spectrum of diagenetic pathways of nacre, that result in different preservation stages described below:

(1) well-preserved, iridescent nacre composed of polygonal tablets, ca. 2–3 μm in diameter, 0.3-μm thick. Original, granular nanostructure of aragonite tablets still discerned (such an exceptional state of preservation was previously described only from aragonite lagerstätten; see [39,66]; Fig 2K-arrows). Closely aligned, flat tablets form successive parallel layers. In cross section, tablets are stacked one above the other and create columns perpendicular to the shell surface (with small overlap between the neighboring columns) (Figs 2G–2N and 6A).
(2) altered nacreous layer with the effects of dissolution most apparent in the direction normal to the nacre lamellae between adjacent columns, where dissolution of the tablet edges successively decreases the diameter of the aragonite tablets. Less prominent alterations are observed in the horizontal plane (upper- and lowermost parts of single tablets). Tablets gained slightly lenticular shape, some still connected by mineral bridges. The areas of dissolution coincide with the distribution of organic matrix in the primary skeleton; spaces between columnar stacks of tablets, left by aragonite and organic matter, now filled with calcite (Figs 2O, 4E, 4F and 6B).
(3) fragments of columns (stacks of a few superimposed tablets) occurring as aragonite enclaves in calcite crystals (Figs 5B, 5D and 6C).
(4) single laths (thinned tablets) of aragonite that appear embedded within calcite crystals. (Fig 4G and 4H-arrows, Figs 5F, 5G and 6D). The laths preserve the original parallel arrangement of the nacre layers (Fig 5F); columnar pattern still discernible (Fig 5E and 5F-arrows, Fig 6D).
(5) neomorphic calcite compose most of the volume of the studied specimens. The traces of the former microstructure are sometimes recognizable in the form of dark impurities (remnants of aragonite tablets), reflecting the previous arrangement of nacre layers (Fig 5A, 5C-black arrow, Figs 6E and S3C, S3E and S3G); the linear pattern, which was parallel to the surface of the shell in non-altered skeletons, now undulating, sometimes consistent with the shape of newly formed calcite crystals (Fig 5E) but more frequently, it crosses crystal boundaries (Fig 4G-arrows, Fig 5C-black arrow, Fig 6E).
(6) part of the shell transformed into coarse crystals of calcite with clear cleavage planes and crystal boundaries (Figs 6F and S3H); no traces of original microstructure known from other primary preserved cephalopod shells.

Some microstructural features of individual shell layers can be described in terms of the Preservation Index (PI). The PI classification was proposed by Cochran et al. [60] for nacreous and by Knoll et al [67] for nacreous and cross-lamellar layers of fossil mollusks to provide a handy tool to assess the preservation of samples before using them for geochemical measurements. The five stages of the PI classification are based on microstructural features, with PI5 representing excellent microstructural preservation ("nacreous tablets distinct from adjacent layers and well-defined" as in modern *Nautilus*) and PI1 representing poor preservation ("nacreous tablets (…) indistinct and fused with adjacent layers"). The best structurally preserved fragments of nacreous layer in our material (Fig 2L–2N) can be assigned to PI4, but preservation of some other, still distinguishable nacre tablets, is clearly worse than Cochran's PI1 stage (e.g., Fig 5). While the PI classification can be extended and still used as a provisional tool to assess mollusk shell preservation, much finer-scale structural details (such as nanostructural/crystallographic characterization) and biogeochemical criteria must be employed for modern, state-of-the-art, high-resolution geochemical measurements (e.g., methods used for diagenetic assessment of scleractinian corals [68] and echinoderms [69]).

### Aragonite-calcite transformation

In the most general terms, calcite may replace original skeletal aragonite by two major pathways: (i) complete dissolution of aragonite and filling of the resulting space by the crystallization of calcite spar and (ii) gradual dissolution of aragonite and subsequent *in situ* crystallization of calcite along a thin solution film [3]. The latter process meets Folk’s [70] definition of *neomorphism* (“all transformations between one mineral and itself or a polymorph”), but this particular case of replacement (calcium carbonates but different polymorphs) might be more specifically termed *inversion* [70]; see also [3,71,72].

The outcome of this fine-scale aragonite-to-calcite transition process is often the presence of remnants of the original phase. Such primarily oriented relicts of aragonite in neomorphosed shells were considered evidence of fast transformation [73]. Glover and Kidwell [74] (see also [5]) proposed a mechanism by which the surface part of the aragonite is transformed into a more stable polymorph, thus inhibiting further dissolution. Additionally, Dullo and Bandel [28] suggested that when slight dissolution of primary shell is followed by rapid crystallization of calcite, the innermost parts of aragonite crystals can be preserved intact. Such isolation from diagenetic solutions could explain the preservation of aragonite relicts in fossils as old as the Ordovician [14]. Similar entrapment of primary aragonite is well visible in micro-Raman images of the cephalopods studied here, in which aragonite fragments (green) are stuck within calcite crystals (magenta) (Fig 4F and 4I). A relatively fast process of progressive dissolution and subsequent crystallization of calcite may lead to the isolation of the innermost fragments of aragonitic nacre. Different closure timing is reflected in various states of preservation (Fig 6B–6E), starting from columnar stacks of nacre tablets, sometimes still joined in groups of two-three columns, through isolated fragments of single columns and finally, single, extremely thinned nacre tablets (laths of aragonite). In regions where diagenesis reached one of the later stages, the aragonite is completely replaced by coarse crystals of calcite (Fig 6F). In such cases, one may not be able to distinguish microscopically whether this secondary calcite is a product of the replacement of aragonitic nacreous or prismatic layers.

Observations of fossil skeletons of corals and bryozoans [2,75] have suggested that when the neomorphic front migrates along bundles of aragonite fibers, it might preserve the shape and orientation of the crystals (which are transformed into calcite) and the arrangement of layers enriched in organics. Migration of such diagenetic fronts may result in local enrichment of dark, linearly arranged impurities. These were also noted in diagenetically altered shells of cephalopods: Dullo and Bandel [28] found periodic dark bands even in neomorphic blocky calcite and interpreted them as the remains of growth lines of ammonite shell. Conversely, our observations suggest that delicate, brownish banding parallel to the shell surface observed in some sections in transmitted light (Figs 4A, 6E and S3C and S3G) represents linearly arranged, thin aragonite tablets rather than organic-enriched growth layers. Such primary arrangement of the nacre sheets can be maintained if the interlamellar organic matrix is replaced by the mineral (e.g., [58]). Decomposition of organic matrices that would start between vertical stacks of nacre tablets (intertabular organic) and between horizontal layers of nacre (interlamellar) could cause thinning of aragonite tablets (following the mechanism proposed by Hendry et al. [47]). Spaces left by the decomposed organic membranes could be filled with secondary calcite, resulting in preservation of the periodic pattern of nacre layers (Figs 4I, 6B–6D, preservation stages (2), (3) and (4) proposed above).

Dullo and Bandel [28] showed that the nacreous layer in totally calcitized cephalopods can be replaced by vertical (perpendicular to the shell surface), regularly arranged calcitic rods described as pseudostacks. The structures observed in *Cymatoceras*? *patens* ZPAL N.III/224 most likely represent a slightly earlier step in this process: a similar, vertical banding pattern was visualized in the partially altered nacreous layer of the shell (transmitted light, cathodoluminescence and micro-Raman images; Figs 4E, 4F, 5C and 5E and S4C–S4F). In accordance with the previous distribution of the intertabular organic matrix, vertical bands of neomorphic calcite separate wider strips of pristine aragonite (i.e., remnants of nacre columns).

According to Dullo and Bandel [28], nacre may also transform into calcite needles, creating a pseudo cross-lamellar structure. However, this structure was described based only on optical similarity to the cross-lamellar structure of aragonite when observed in thin section, so we do not include it in the nacre diagenesis model presented herein.

### Enigma of aragonite preservation in porous Cretaceous siliceous limestones

Skeletons composed of aragonite generally have rather low preservation potential, but under some conditions, the specimens may be sealed off from the destructive agents and avoid dissolution. The factors that lead to the preservation of aragonite are impermeability of the shales or clays [27,76–78], impregnation with oil [62], or separation between bituminous layers [79]. The chances of aragonite preservation increase in rapid burial, e.g., by density currents or in storm beds [80–83]. The formation of hardground may inhibit burrowers that provide oxygen to the sediment (causing pH reduction), thus stopping the dissolution in the taphonomically active zone (TAZ) [80]. The pristine aragonite shells might be protected by coating of the encrusting biota, e.g., diatoms [84]. Aragonite preservation is also favored in the organic-enriched, mud-dominated low-energy environments. Jordan et al. [82] also suggested a link between the stability of aragonite during early diagenesis (no dissolution) and anoxic episodes that can shorten the residence time of the skeletons in TAZ.

When one of those conditions occurs, it might lead to preservation of diverse assemblages of unaltered aragonite fauna. More frequently, none of these conditions is fulfilled, and aragonitic remains are completely replaced or dissolved. However, a growing number of reports show that preservation of skeletal aragonite is not an “all-or-nothing” phenomenon (see [85]). Some “exceptionally preserved” aragonite fossils appear to have a significant portion of secondary calcite (e.g., [38]), whereas some severely diagenetically altered specimens reveal relics of primary microstructure [14,73,75,86]. The cephalopod fossils studied here provide an insight into the several intermediate steps of the aragonite-to-calcite transformation (see “Preservation of fossil cephalopod shells”; Fig 6). Based on our observations, it seems that the pattern and rate of diagenesis can be locally influenced by a combination of factors acting at the microscale (causing differences in preservation, even within a single shell; Figs 4, 5 and S3), sometimes regardless of unfavorable skeletal mineralogy or the lithology of the host rock.

The examined aragonite fragments of the shells were found in siliceous limestones with high porosity [19,20], which should have promoted rapid dissolution and the removal of the skeleton from the fossil record. For example, in British Cretaceous chalk the aragonite is “totally absent” [18]. Moreover, in the studied Cretaceous sections the majority of formerly aragonitic shells was dissolved or transformed into calcite, but some cephalopods preserved remnants of pristine skeleton. A similar case of selective preservation of cephalopod shells (goniatites and orthocone nautiloids) was observed by Hallam and O’Hara [78] in the Carboniferous of Scotland; co-occurring originally aragonitic gastropods were more altered and composed of a chalky mixture of calcite and aragonite. The two groups of factors that may drive differential preservation of mollusk shells with the same mineralogy are discussed below.

#### Shell size and porosity

The size of the specimen is considered one of the main factors determining selective preservation of aragonite fossils [87]; small specimens are more delicate and prone to fragmentation and dissolution [88]. A laboratory study of carbonate skeletons placed in acid baths revealed that the dissolution rate is strongly dependent on the surface area-to-weight ratio (specimens with higher ratios dissolved faster) [89]. Additionally, experiments with buried skeletons showed that thinner shells are relatively more vulnerable to dissolution [90,91]. The higher porosity of the skeleton (natural or resulting from bioerosion) and thus the larger surface available for dissolution also increases the rate of diagenesis [92]. Surprisingly, these factors may have a greater impact on dissolution than mineralogical differences and compact aragonitic shells may have the same or even greater resistance than thin and porous calcitic shells [89]. The smallest and thinnest shells have the least chance of surviving in the fossil record [88,93]. Finally, the thickness, compactness of the cephalopod shell and the stiffness of its nacre layer (see [57,94]) could facilitate its preservation in comparison to more porous skeletons co-occurring in the same layers.

Dissolution of smaller aragonite remains may locally increase the alkalinity, pH and aragonite saturation of the pore water and consequently slow the dissolution of larger shells [82,95]. Such “self-buffering” of the pore waters by dissolution of fragmented shells was proposed as a mechanism of aragonite preservation in the fossil record [91,93]. A similar mechanism could also explain the preservation of aragonite fragments of relatively large and thick shells of nautilids and ammonites described herein that are preserved in deposits containing abundant casts and moulds of small aragonitic-shelled organisms. Accordingly, dissolution of smaller carbonate skeletons would increase local pH and infacilitate the preservation of aragonite. The voids between the aragonite lining that covers the mould of *Baculites* (Fig 1D) and the host rock indicate that the dissolution occurred after lithification of the sediment. The progressive dissolution of the outer part of the shell could at some point change the chemistry of the solution and inhibit the next steps of diagenesis.

#### Shell microstructure and organic matrix content

Considering its relatively higher solubility, the aragonite should be altered much faster than calcite (“mineral controlled diagenesis” [13]), but if the solution is undersaturated with respect to both polymorphs of CaCO_3_, the microstructure has a greater impact on the rate of skeleton dissolution than its mineralogy [91,96]. Accordingly, some of the aragonitic microstructures appeared to be more resistant to alteration than calcitic ones [97]. Observations of rare fossil cephalopods preserved with all three mineral layers of the shell have further suggested variations in the diagenesis of adjacent aragonite units of different microstructure [39,98]. The difference in preservation of two types of aragonite microstructures was recently noted by Knoll et al. [67] in mollusks from the Upper Cretaceous deposits of the Gulf Coastal Plain and the Western Interior Seaway, US. The observed difference between the proportions of "poorly preserved" (Preservation Index scale) specimens composed of nacreous vs. cross-lamellar layers suggested that susceptibility to diagenesis depends on skeletal microstructure. The differential vulnerability to deterioration of crossed lamellae in gastropods and bivalves shows that even slight dissimilarities in skeletal architecture can result in different states of aragonite preservation. In our specimens, aragonite was only found in cephalopods and in the form of nacre, suggesting peculiar properties of this shell layer.

The presence of interfacial organic phase and mineral bridges between tablets makes the nacreous microstructure stiffer [57], which might promote endurance through the first steps of diagenesis/postmortem transport. The relatively greater durability of nacre was previously demonstrated in experiments involving the dissolution of bivalve shells of different mineralogy and microstructure [97]. It was shown that nacre loses weight much more slowly than calcite foliae and cross-lamellar microstructures or homogenous aragonite. In contrast, the prismatic (both aragonitic and calcitic) microstructures appeared to be the most vulnerable to degradation (*ibidem*), supporting our observations on cephalopods.

The microstructure may control the rate and geometry of skeletal neomorphism [54,86]. The degree and direction of changes depend on crystal size, shape and orientation to the dissolved outer surface of the shell [3,97]. The resistance of different microstructures has been suggested to be inversely proportional to skeletal organic content [74], but specific types of microstructure also vary in the composition and spatial delineation of organic matter, which might cause differential diagenesis of mineralogically similar fossils [54,89,91]. Likewise, our observations suggest that the orientation and spatial relations between crystals and interlamellar membranes can have a greater influence on skeleton preservation then the amount of organic matter.

The lack of a prismatic layer and the preservation of nacre within the same shell observed herein may partially result from differences in the orientation of crystallites. In the prismatic layer, crystallites are arranged perpendicular to the surface of the shell, so all are in direct contact with the dissolved surface. Moreover, prisms are surrounded by vertical organic membranes that are also easily exposed to percolating solutions. The decay of organics might facilitate access to all crystal faces [97], accelerate dissolution and splitting of the prismatic layer.

On the other hand, observations of the Ordovician phosphatized mollusks from the Maquoketa Formation (USA) showed that the interlamellar organic matter of the nacre might be resistant to diagenetic alteration [59]. Mutvei [98] suggested the influence of interlamellar membranes on the preservation of the nacreous layer in cephalopods from the Pennsylvanian Buckhorn Asphalt Lagerstätte. The hypothesis about the protective role of organics was also supported by observations of Cretaceous ammonites [99], in which fragments of original nacre were associated with previously damaged regions of the shell. The higher concentration of organic matter in regenerated parts of the skeleton was considered responsible for aragonite preservation.

The preservation of organic-enriched nacreous layer may be explained by the mechanism proposed by Kennedy and Hall [16] for well-preserved aragonite fossils of Cretaceous Gault Clay. Accordingly, the organic matrix of the shell disintegrates into soluble amino acids with charged and hydrophilic amine (-NH_2_) and carboxyl (-COOH) groups but with nonpolar or hydrophobic side-chains (R-group). The amine and carboxyl groups are attracted to CO_3_^2-^ and Ca^2+^ ions on the surface of calcium carbonate crystals “where the termination of the crystal structure resulted in unsatisfied ionic charges” (*ibidem*). Consequently, the newly formed monolayer of amino acid molecules covers the aragonite crystals with the hydrophobic R-groups, creating a waterproof barrier.

We suggest that nacreous microstructure might have a preference to preserve by the process proposed by Kennedy and Hall [16]. In the case of horizontally (parallel to the shell surface) arranged laminae of nacre, the innermost parts might be protected by superimposed layers of aragonite tablets and organics. The formation of a hydrophobic surface on one of the nacreous laminae could explain the preservation of thin aragonite lining on the ammonite moulds, where the outer part of the shell and accompanying fossils were completely dissolved (Figs 2 and 3).

## Conclusions

The spectrum of alternation observed in Cretaceous skeletons leads us to extend the existing model of cephalopod nacre diagenesis with several intermediate steps of aragonite-to-calcite transformation. The primary aragonitic shells preserved as (i) internal mould covered with unaltered or only slightly changed nacreous layer, (ii) calcitized skeletons that preserved remnants of aragonite nacre and (iii) completely recrystallized shells composed of blocky calcite. The selective preservation of aragonite in only the nacreous layer of ammonites and nautilids most likely results from the combination of different factors that favored conservation of this generally metastable polymorph of CaCO_3_. The remnants of nacre tablets found in recrystallized shells could be entrapped in secondary crystals of calcite during the rapid process of aragonite dissolution and the subsequent crystallization of calcite. The pattern of diagenetic alterations of the shell reflected the distribution of the skeletal organic matrix, so the primary arrangement of the aragonite tablets is still discerned. Here, a significant role in the preservation of aragonite fossils is also assigned to the skeletal microstructure, which is composed of intercalating sheets of aragonite and organic membranes. The decay of amino acid-enriched organic components could facilitate the formation of a hydrophobic adsorbed layer (adlayer) that inhibits dissolution of the underlying aragonite and leads to preservation of the thin iridescent lining observed on the cephalopod moulds.

Contrary to previous suggestions (e.g., [74]), the organic-enriched nacreous layer is preserved better than accompanying fossils with different structures. The organic matter, which plays a key role in the formation of biomineral skeleton, appeared to also be important for its postmortem history. Upper Cretaceous siliceous limestones of eastern Poland and western Ukraine can no longer be considered as lacking the original aragonite. The unaltered parts of the aragonite fossils found in these deposits may even become a source of geochemical data.

## Supporting information

S1 Fig Locality map with indication of the Krasnobród and Potelych sections mentioned in the text (stars). The extent of Upper Cretaceous deposits (both cropping out and under Quaternary cover) is marked with green. Comparative materials illustrated in the paper (Fig 1) were collected in the outcrops at Nasiłów and Piotrawin marked with black squares (for detailed description of the outcrops see [22] and [100] respectively). (Map simplified after [101]).
(TIF)

S2 Fig X-ray diffractograms of pulverized fragments of iridescent shells of ammonite *Baculites* sp. **(A) and nautilid *Eutrephoceras vastum* (B), both specimens from Krasnobród, indicating presence of aragonite (and calcite) and reference samples of synthetic aragonite (C) and calcite (D).** A- specimen ZPAL Am. 12/1375; B–specimen ZPAL N. III/219.
(TIF)

S3 Fig Nacre relics in diagenetically altered shell of nautilid *Cymatoceras*? *patens*. (A) Thin section of the shell wall in transmitted light and (B) CL images that suggest different state of preservation of the lower, innermost (dark, arrow) and middle part of the shell (orange). Bright orange luminescence in originally aragonitic specimens is usually considered as an effect of diagenesis, whereas the lack of a luminescence might indicate less altered areas of the skeleton. Surprisingly, micro-Raman mapping of CaCO_3_ polymorph distribution (D), of the same section shows mixture of aragonite and calcite (green- aragonite, magenta–calcite) in both parts of the shell. Dashed frame in (A) indicate regions enlarged in (G) and (C). (C) Close-up on the section, the white frame deliminates region shown in (E). (E-F) SEM micrographs of thin section; Aragonite detected by micro-Raman corresponds to the inclusions of nacre surrounded by secondary calcite, visible in SEM images (E) and (F) (arrows). Despite the difference between the lowermost and middle part of the shell revealed by CL imaging, both regions observed in SEM (F) and in enlarged image of thin section (G, arrows) contain laths of aragonite, i.e., remains of nacreous layer; (H) thin section of the adjacent fragment of the same shell where aragonitic microstructure transformed into blocky calcite with clear cleavage lines. (A-H) Specimen ZPAL N.III/224.
(TIF)

S4 Fig Transmitted light (left column) and cathodoluminescence images (right column) of the shell wall and septum of nautilid *Cymatoceras*? *patens*. **(**A,B) Fragment of the shell wall and septum and (C-F) close-ups of the septum. The inner (lower) part of the septum (arrows) in TLM and CL images showed alternating dark and bright bands arranged perpendicular to the septal plane. The bright luminescent bands in CL correspond to calcite that filled the space between columns of nacre. The dark (less altered) regions in CL correlate with brown areas in transmitted light images, which in SEM were recognized as remnants of columns of nacre tablets (Fig 4D). This interpretation of the pattern observed under CL was confirmed by micro-Raman images, showing aragonite (green) remnants of nacre tablets arranged in vertical stacks and separated by calcite (magenta); compare with Fig 3F. (A-F) Specimen ZPAL N.III/224.
(TIF)

S5 Fig Mg/Ca and Sr/Ca ratios of aragonitic and calcitized parts of *Cymatoceras*? shell. (A-C) Thin-sectioned fragment of the septum with darker, laminated zone (remnants of nacreous layer) that interfinger with strongly altered region composed of blocky calcite (A, transmitted light; B, fragment of A in polarized light); (C) micro-Raman image show that small enclaves of aragonite (green) are entrapped in calcite crystals (magenta). Circled numbers (1–5) correspond to points of EDS analysis. The higher concentration of strontium was detected only in the region interpreted as aragonite remnants of nacreous layer. The same region has also slightly higher concentrations of magnesium. (A-C) Specimen ZPAL N.III/224.
(TIF)

S1 Tab Inventory numbers, localities, and geological age of the specimens treated and illustrated in the present study. (TIF)
