# Supplementary figures and images for: From pristine aragonite to blocky calcite: Exceptional preservation and diagenesis of cephalopod nacre in porous Cretaceous limestones
Source: PLoS One. 2018 Dec 19;13(12):e0208598. doi: 10.1371/journal.pone.0208598 (PMC6300390; doi:10.1371/journal.pone.0208598)

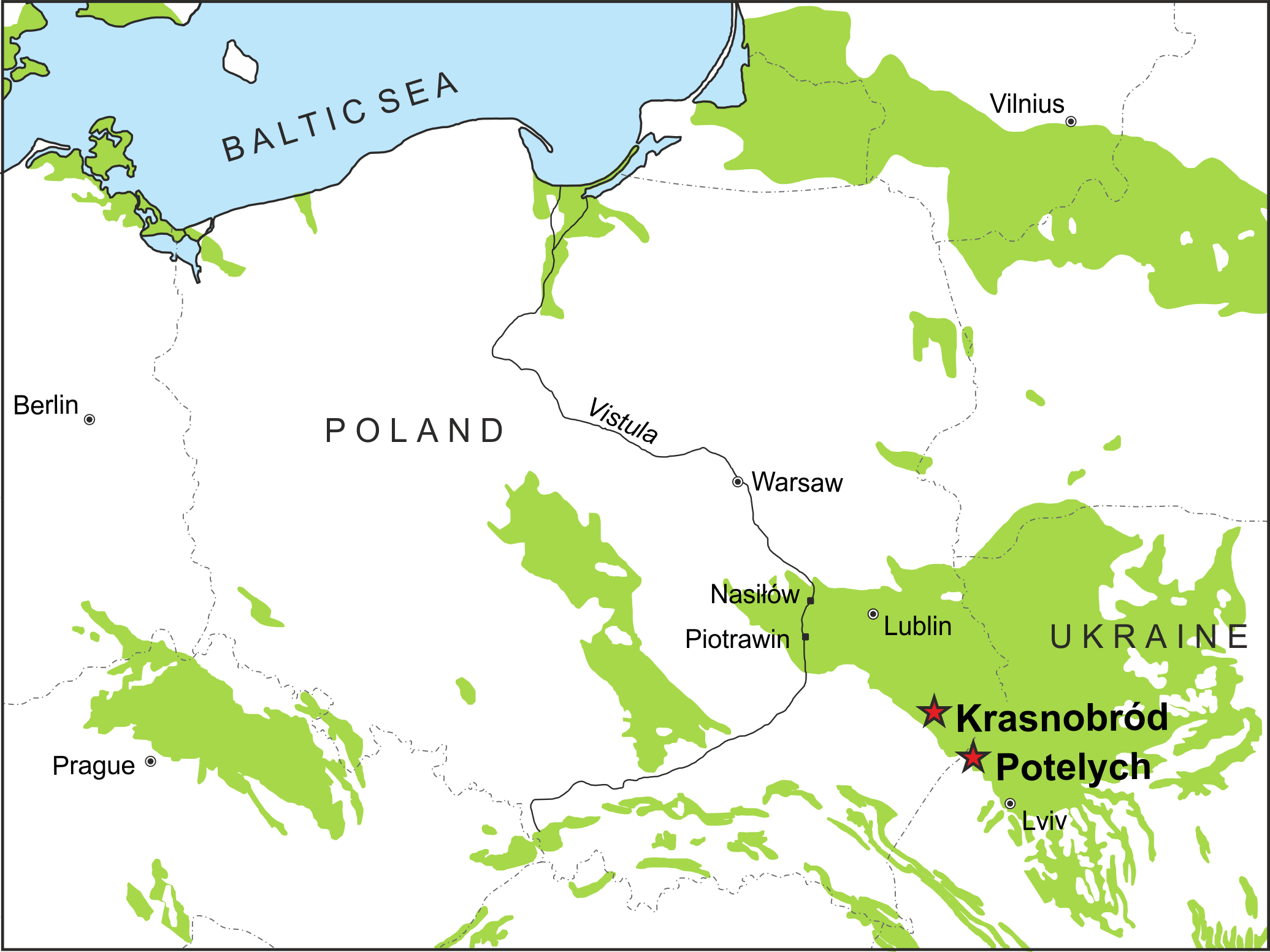

Supplement: S1 Fig — The extent of Upper Cretaceous deposits (both cropping out and under Quaternary cover) is marked with green. Comparative materials illustrated in the paper (Fig 1) were collected in the outcrops at Nasiłów and Piotrawin marked with black squares (for detailed description of the outcrops see [22] and [100] respectively). (Map simplified after [101]). (TIF) [file pone.0208598.s001.tif]

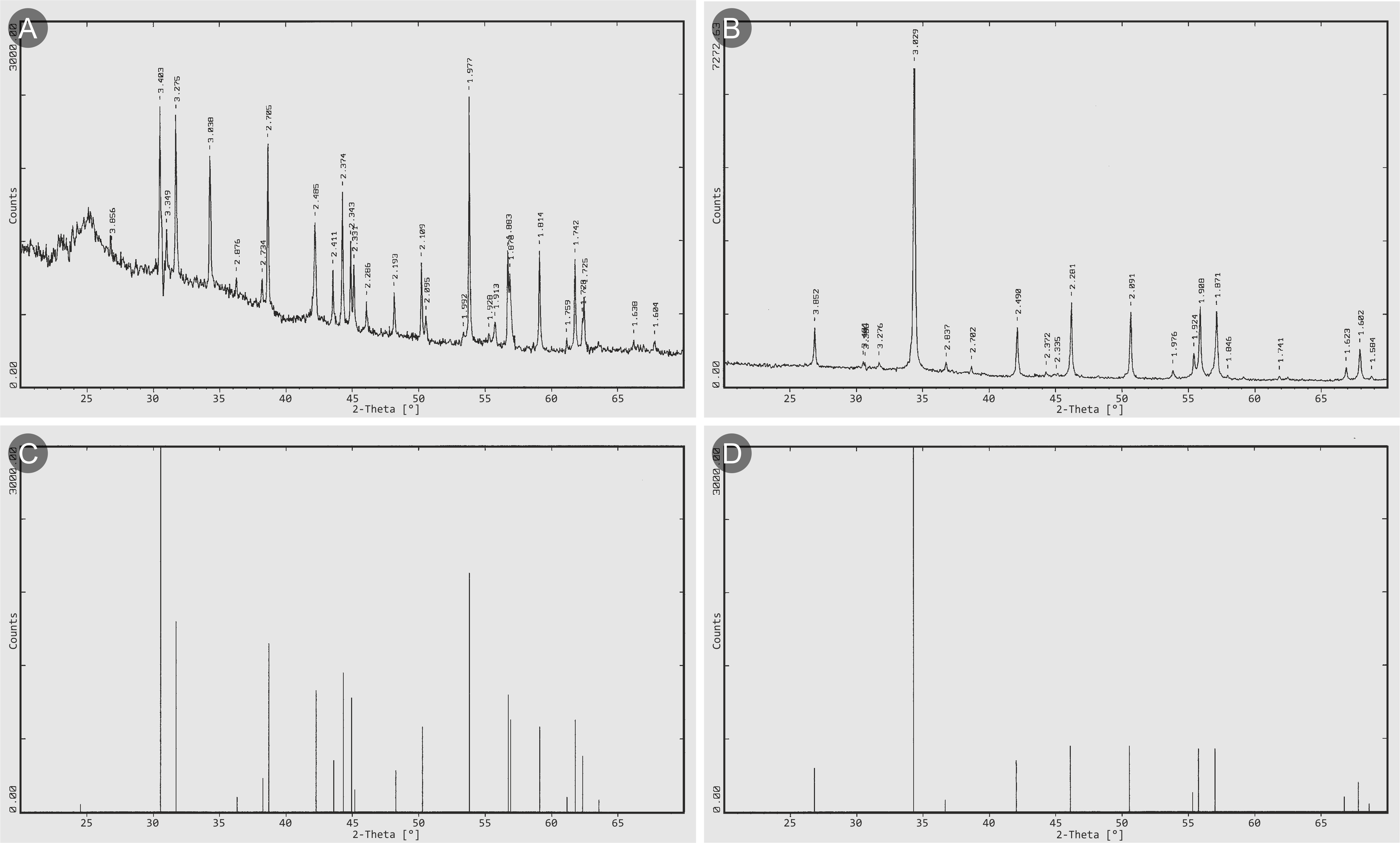

Supplement: S2 Fig — (A) and nautilid Eutrephoceras vastum (B), both specimens from Krasnobród, indicating presence of aragonite (and calcite) and reference samples of synthetic aragonite (C) and calcite (D). A- specimen ZPAL Am. 12/1375; B–specimen ZPAL N. III/219. (TIF) [file pone.0208598.s002.tif]

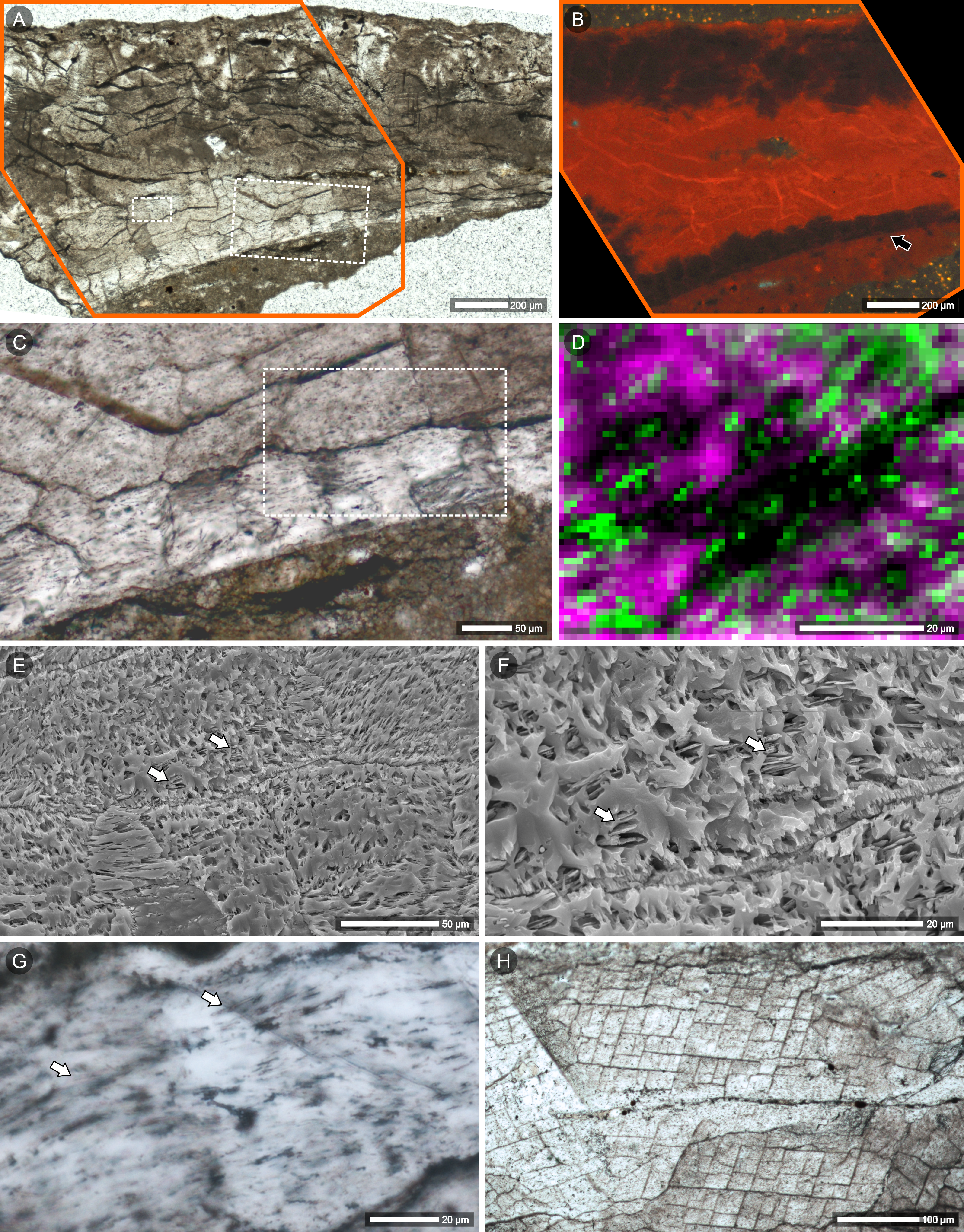

Supplement: S3 Fig — (A) Thin section of the shell wall in transmitted light and (B) CL images that suggest different state of preservation of the lower, innermost (dark, arrow) and middle part of the shell (orange). Bright orange luminescence in originally aragonitic specimens is usually considered as an effect of diagenesis, whereas the lack of a luminescence might indicate less altered areas of the skeleton. Surprisingly, micro-Raman mapping of CaCO3 polymorph distribution (D), of the same section shows mixture of aragonite and calcite (green- aragonite, magenta–calcite) in both parts of the shell. Dashed frame in (A) indicate regions enlarged in (G) and (C). (C) Close-up on the section, the white frame deliminates region shown in (E). (E-F) SEM micrographs of thin section; Aragonite detected by micro-Raman corresponds to the inclusions of nacre surrounded by secondary calcite, visible in SEM images (E) and (F) (arrows). Despite the difference between the lowermost and middle part of the shell revealed by CL imaging, both regions observed in SEM (F) and in enlarged image of thin section (G, arrows) contain laths of aragonite, i.e., remains of nacreous layer; (H) thin section of the adjacent fragment of the same shell where aragonitic microstructure transformed into blocky calcite with clear cleavage lines. (A-H) Specimen ZPAL N.III/224. (TIF) [file pone.0208598.s003.tif]

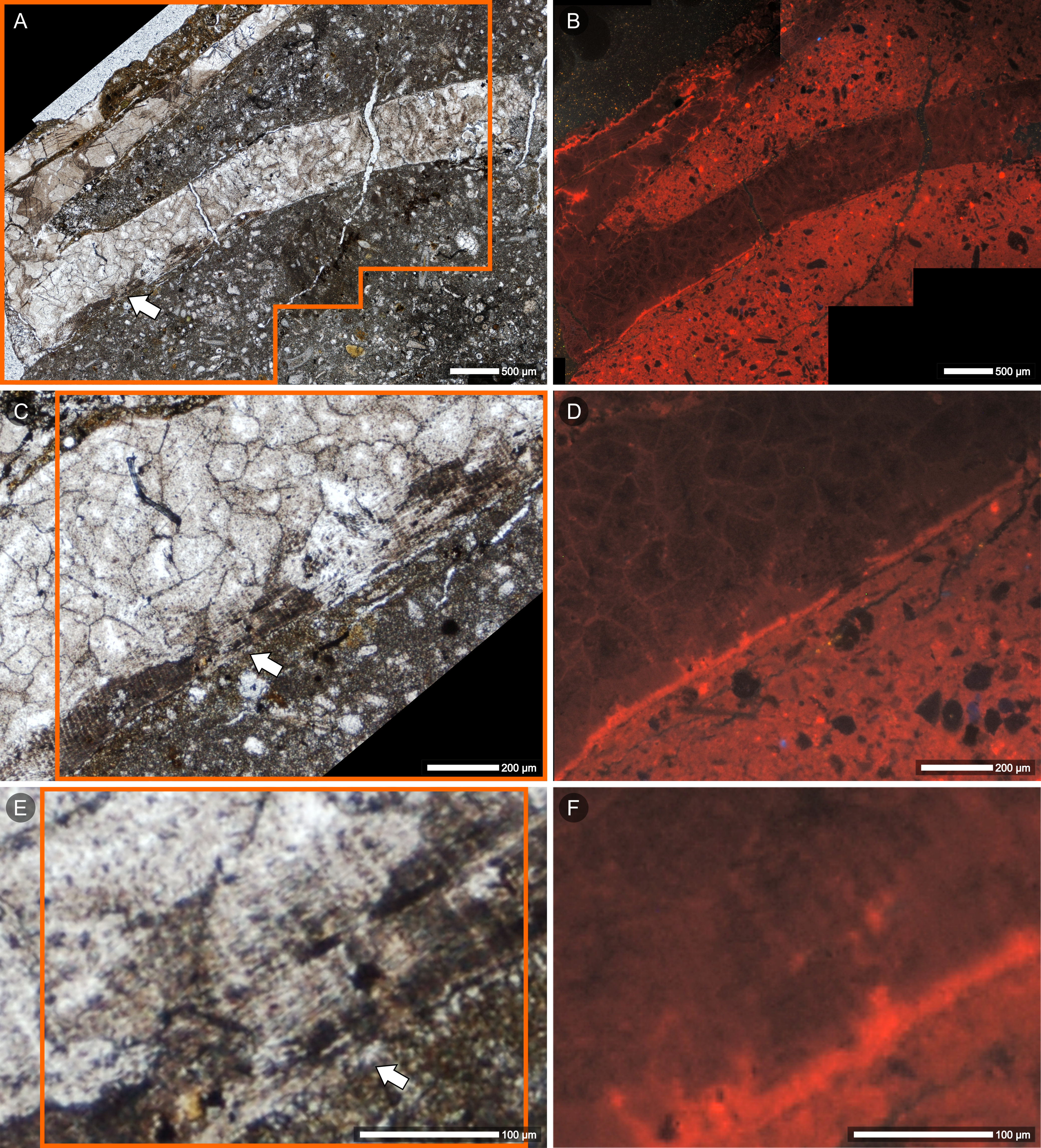

Supplement: S4 Fig — (A,B) Fragment of the shell wall and septum and (C-F) close-ups of the septum. The inner (lower) part of the septum (arrows) in TLM and CL images showed alternating dark and bright bands arranged perpendicular to the septal plane. The bright luminescent bands in CL correspond to calcite that filled the space between columns of nacre. The dark (less altered) regions in CL correlate with brown areas in transmitted light images, which in SEM were recognized as remnants of columns of nacre tablets (Fig 4D). This interpretation of the pattern observed under CL was confirmed by micro-Raman images, showing aragonite (green) remnants of nacre tablets arranged in vertical stacks and separated by calcite (magenta); compare with Fig 3F. (A-F) Specimen ZPAL N.III/224. (TIF) [file pone.0208598.s004.tif]

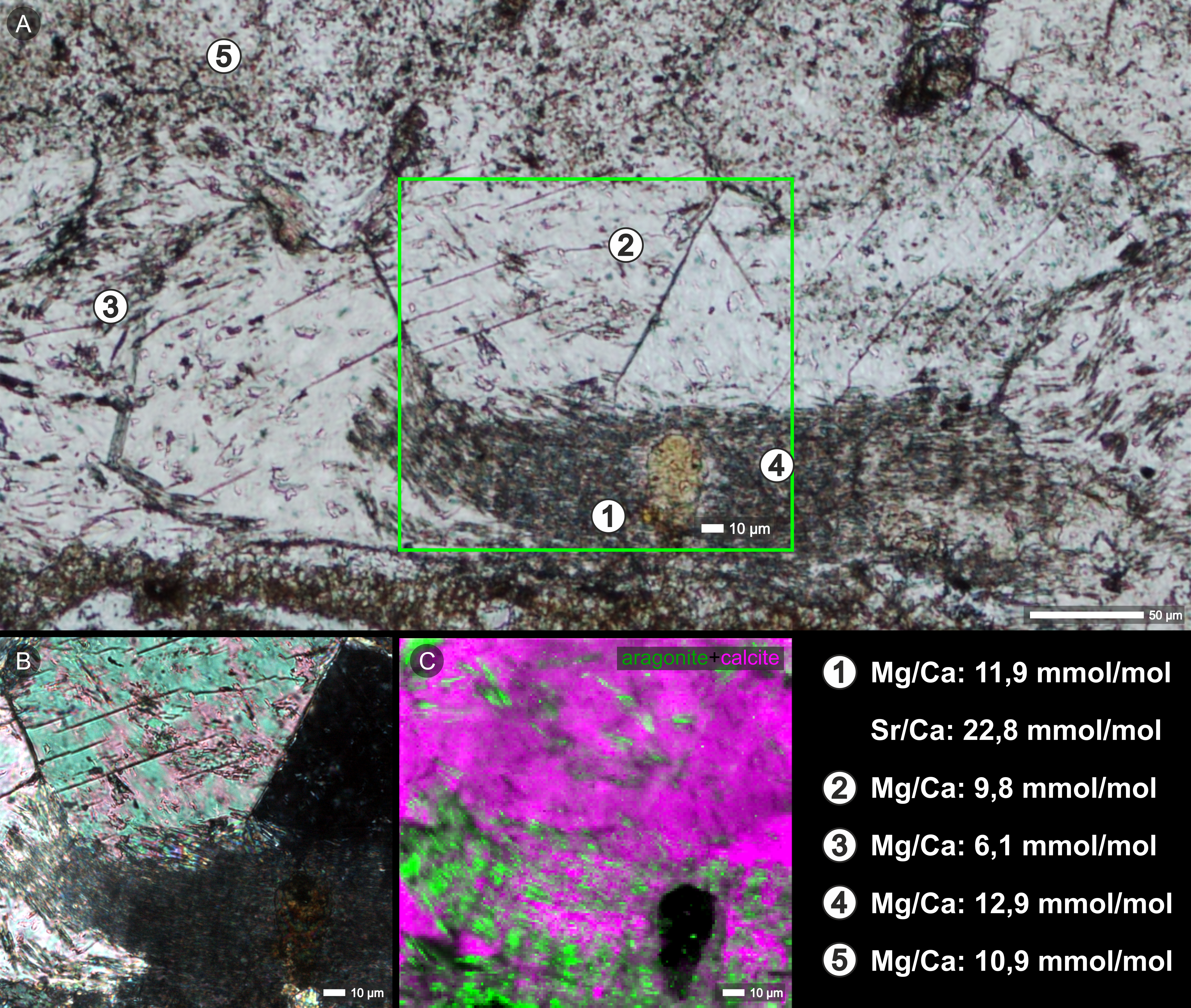

Supplement: S5 Fig — (A-C) Thin-sectioned fragment of the septum with darker, laminated zone (remnants of nacreous layer) that interfinger with strongly altered region composed of blocky calcite (A, transmitted light; B, fragment of A in polarized light); (C) micro-Raman image show that small enclaves of aragonite (green) are entrapped in calcite crystals (magenta). Circled numbers (1–5) correspond to points of EDS analysis. The higher concentration of strontium was detected only in the region interpreted as aragonite remnants of nacreous layer. The same region has also slightly higher concentrations of magnesium. (A-C) Specimen ZPAL N.III/224. (TIF) [file pone.0208598.s005.tif]

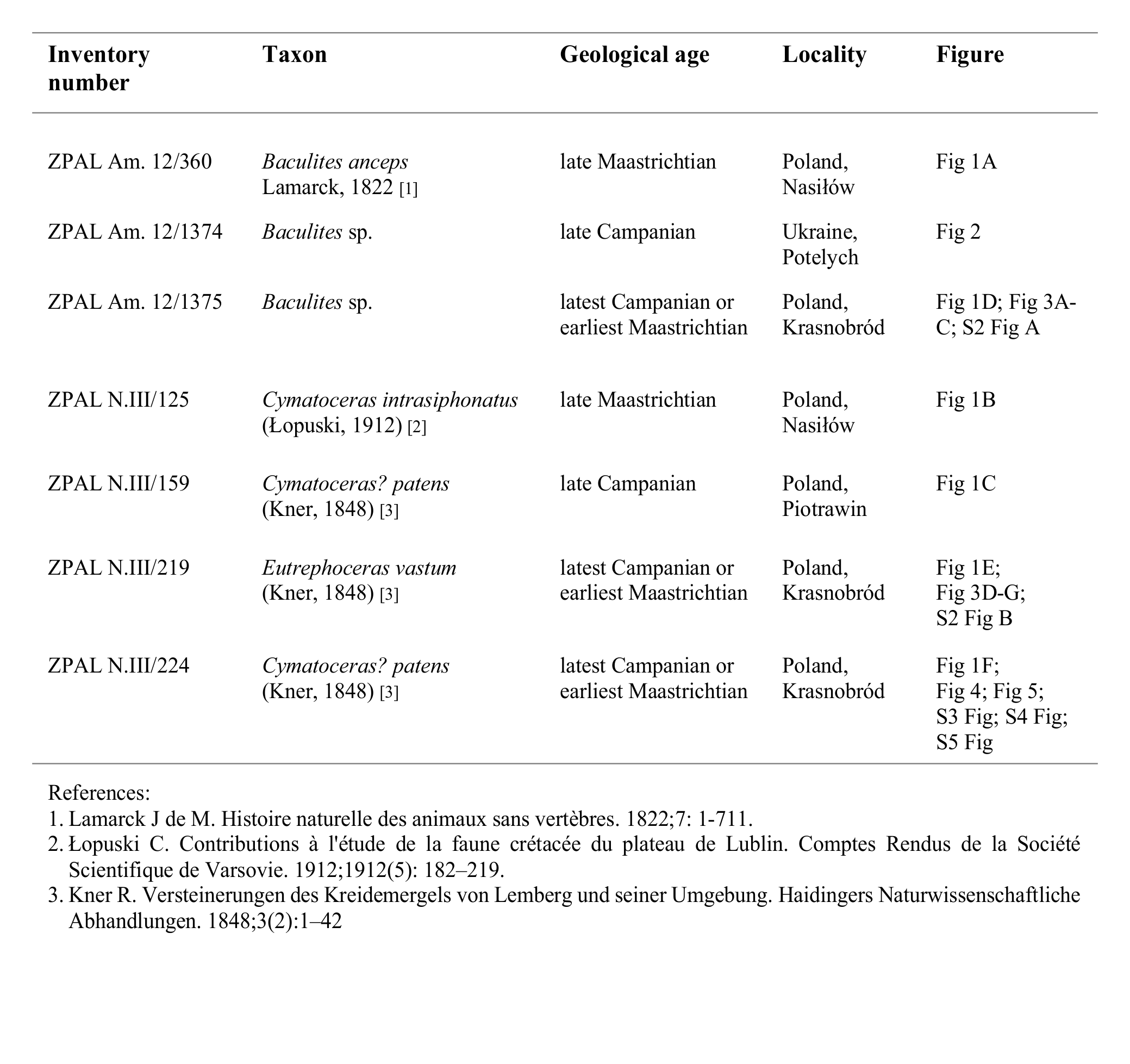

Supplement: S1 Tab — (TIF) [file pone.0208598.s006.tif]
